# Simple and efficient way to detect small polymorphic bands in plants

**DOI:** 10.1016/j.gdata.2015.06.006

**Published:** 2015-06-11

**Authors:** Manu Kumar, Seong Ryong Kim, Prabodh Chander Sharma, Ashwani Pareek

**Affiliations:** a Department of Life Science, Sogang University, Seoul 121-742, South Korea; b Crop Improvement Division, Central Soil Salinity Research Institute, Karnal 132 001 (Haryana) India; c Stress Physiology and Molecular Biology Laboratory, School of Life Science, Jawaharlal Nehru University, New Delhi 110067, India

**Keywords:** SSR, simple sequence repeat, TBE, tris borate ethylenediaminetetracetic acid, TEMED, tetramethylethylenediamine, *Oryza sativa*, *Brassica juncea*, Microsatellite, SSR, PAGE, Silver staining

## Abstract

There are many ways to detect polymorphism. In this study we use the microsatellite markers to detect the polymorphism for the salt tolerance. This method has been successfully conducted in *Oryza sativa* and *Brassica juncea*. The results are reproducible. In contrast to previous methods, our method is simple and quite accurate for detecting the polymorphic bands. In this study instead of using agarose gel and ethidium bromide staining, we used non-denaturing polyacrylamide gel and a low-cost improved method for silver staining when we compare it to 11 other methods for their ability to detect simple sequence repeat polymorphisms as small as 50 bp in denaturing polyacrylamide gels. All methods detected the same alleles and banding pattern. However, important differences in sensitivity, contrast, time consumption and background were observed.

## 1 Introduction

In plant breeding, conservation and evolutionary studies, molecular markers such as simple sequence repeats (SSRs) or sequence-tagged microsatellite sites (STMS), are widely used (e.g., genome mapping, marker assisted selection). Efficient methodology is required for mass genotyping [1]. Uses of the radioactive or fluorescent labelled nucleotides are the most efficient way to visualize single strand DNA in polyacrylamide gels. These procedures are expensive, time consuming and require special facilities and it makes them impracticable for most of the tropical countries where sophisticated infrastructures are lacking [2].

The silver-staining of proteins has been used for a wide variety of physical and biological analyses in polyacrylamide gels. In last decade, it has been applied in polyacrylamide gels to detect nucleic acid as silver ions bind to the bases and then under alkaline conditions it can be selectively reduced by formaldehyde. For detecting DNA in PCR-single strand conformation polymorphism analysis, silver staining of nucleic acid is widely used. Many other alternative methods to silver staining for DNA detection have been described previously. However, most of them are not rapid enough [3] because of time consuming steps and involve the changing of solutions repeatedly. It is hard to establish a highly output staining method. Although many simplified methods have been reported before [4], [5], [6], [7], [8], [9], [10], [11], [12], [13], [14], they still lack in sensitivity and/or efficiency.

The main objective of this study was to evaluate and standardize a new low-cost method for detecting polymorphism using silver staining using rice (*Oryza sativa*) and Indian mustard (*Brassica juncea*) as a model system. We compared sensitivity of this procedure to the 11 other commonly used procedures [4], [5], [6], [7], [8], [9], [10], [11], [12], [13], [14] and optimized the reaction conditions for detection of polymorphism by using denaturing polyacrylamide gels.

## 2 Materials

### 2.1 Reagents and equipment

#### 2.1.1 Genomic DNA isolation

CTAB buffer, Microfuge tubes, Mortar and Pestle, Liquid nitrogen, Microfuge, 70% Ethanol (ice cold), Isopropanol, 60 °C water bath, Chloroform: Iso-amyl alcohol (24:1), Water (sterile), Agarose, 6 × Loading buffer, 1 × TBE solution, Agarose gel electrophoresis system, Ethidium bromide solution.

##### 2.1.1.1 Elution buffer 100 ml

2.0 g CTAB (Hexadecyl trimethyl-ammonium bromide), 28.0 ml 5 M NaCl, 4.0 ml 0.5 M EDTA pH (8.0), 10.0 ml 1 M Tris-cl (pH 8.0), 1% (*v*/*v*) 2-Mercaptoethanol, 1% (*w*/*v*) polyvinyl-pyrrolidone (PVP, Mw 10,000). Adjust all to pH 5.0 with HCL and make up to 100 ml with H_2_O.

##### 2.1.1.2 1 M Tris pH 8.0

Dissolve 121.1 g of Tris base in 800 ml of H_2_O. Adjust pH to 8.0 by adding 42 ml of concentrated HCL. Allow the solution to cool to room temperature before making the final adjustments to the pH. Adjust the volume to 1 l with H_2_O. Sterilize using an autoclave.

#### 2.1.2 Polymerase chain reaction using microsatellite markers

10 mM Tris–HCl (pH 8.3), 50 mM KCl, 1.5 mM MgCl_2_, 200 μM dNTP, 0.4 μM 20 mer primer, 1 unit Taq DNA polymerase, 20 ng template DNA.

### 2.2 Non-denaturing polyacrylamide gel electrophoresis for the separation of the bands

#### 2.2.1 Buffer and solutions

Acrylamide: bisacrylamide (29:1) (% *w*/*v*), Ammonium persulfate (10% *w*/*v*), Ethanol, KOH/methanol, 6 × Gel-loading buffer, 5 × TBE Electrophoresis buffer, TEMED.

#### 2.2.2 Special equipment

Electrophoresis apparatus, glass plates, combs and spacers, gel-sealing tape, micropipette with drawn-out plastic tip, petroleum jelly and syringe.

### 2.3 Detection of the polymorphism in non-denatured polyacrylamide gel by silver staining

Acetic acid (3% *v*/*v*), Developer — Dissolve 30 g of sodium carbonate in a final volume of 1 l of distilled H_2_O. Ethanol (10% *v*/*v*), Formaldehyde (37% *v*/*v*), Nitric acid (0.7% *v*/*v*), Silver nitrate (0.2% *w*/*v*) freshly prepared and Gel scanner.

## 3 Procedure

### 3.1 Legend







The study comprised of four main steps (Fig. 2):

1. Genomic DNA isolation.
2. Polymerase chain reaction using microsatellite markers.
3. Non-denaturing polyacrylamide gel electrophoresis for the separation of the bands.
4. Detection of the polymorphism in polyacrylamide by silver staining.

#### 3.1.1 Genomic DNA isolation

Extraction of DNA from 10 DAG seedlings was isolated from salt tolerant and salt sensitive wild type variety of *O.* *sativa* and *B.* *juncea* as described by [15], [16].

#### 3.1.2 Polymerase chain reaction using microsatellite markers

: *Annealing temperature* (*Tm*) *can be adjusted according to primer set.*

Amplification reactions were carried out in a volume of 20 μl. Reaction mixtures contained 10 mM Tris–HCl (pH 8.3), 50 mM KCl, 1.5 mM MgCl_2_, 200 μM each dNTP, 0.4 μM of 20-mer primer, 1 unit Taq DNA polymerase and approximately 20 ng of template DNA. The amplification was carried out using a thermocycler. The thermal cycler was programmed to one cycle of 4 min at 94 °C for the initial strand separation, followed by 35 cycles of 30 s at 94 °C for denaturation, 1 min at 60 °C and 72 °C for primer extension. Finally, one cycle of 10 min at 72 °C was used for the final extension, followed by storing at 4 °C.

: *Percentage of the polyacrylamide gel depends on the size of the fragment of DNA.*

#### 3.1.3 Non-denaturing polyacrylamide gel electrophoresis for the separation of the bands

1. Wash the glass plates and spacers well first in tap water then in deionized water. Hold the plates from the edges to save the surface of the glasses. Rinse the plates with ethanol and let them dry.
2. Assemble the glass plate with spacer.

: *Apply the agarose gel or wrap the tape around it except on the top so that gel liquid does not leak out.*

3. Taking into account the size of polymorphic bands, the size of glass plate and the thickness of the spacers 6% of PAGE (50 ml) were made by using 10 ml 30% acrylamide + 1% N,N-Methylenebisacrylamide, 29.965 ml H_2_O, 10 ml 5 × TBE and 350 μl of 10% ammonium persulfate.
4. Add 17.5 μl TEMED for 50 ml of acrylamide: bis solution to polymerize the gel.
5. Pour the casting solution into the glass plate carefully.

: *Be careful*, *air bubble under the teeth of the comb should be avoided*, *immediately insert the comb into the gel. Make sure the gel does not leak.*

6. Allow the acrylamide to polymerize for 30 min at room temperature.
7. After polymerization, remove combs very carefully and rinse the wells with 1 × TBE.
8. Attach the gel to electrophoretic tank using the clamps built in the apparatus. Fill the electrophoretic tank with the 5 × TBE. Use pipette to flush out wells once more.

: *Make sure that the wells you are using are absolutely clean from gel pieces and the well should be intact to get a better result.*

9. 6 × gel loading buffer was made by 0.25% bromophenol blue, 0.25% xylene cyanol FF and 30% glycerol in water.
10. 20 μl PCR products were used for loading with 3 μl of 6 × dye.
11. Run the gel on 50 V till dye comes closer to bottom.

: *Running the gel to lower voltage is very important for the bands to be linear.*

#### 3.1.4 Detection of the polymorphism in non-denatured polyacrylamide gel by silver staining

: *Be neat and clean*, *silver nitrate will stain skin*, *cloths*, *walls*, *floors and anything that comes in contact with it. Powder free gloves should be used. Container for gel should be large enough so that gels can float freely.*

12. After electrophoresis, place the gel very carefully (still attached to one plate) in a plastic tray reserved for silver staining.

: *Do not touch the surface of the gel at any time it can cause background staining from fingerprints*, *use gloves.*

13. Rinse the gel twice with distilled H_2_O to remove electrophoresis buffer and gel pieces. During rinsing, the gel will float freely; glass plate can then be removed.
14. Fix the gel in 10% ethanol by shaking gently for 10 min on the shaker. Remove the 10% ethanol by suction and repeat the process 3 times.

: *3 times washing is very important to make background around the gel clear.*

: *Gel can be left at this stage for several hours in 10% ethanol.*

15. Add just enough 0.7% nitric acid to cover the gel. Shake the gel gently on the shaker for 6 min. Remove the nitric acid by suction and rinse the gel 2 times with distilled H_2_O.
16. Add just enough 0.2% silver nitrate to cover the gel. Shake the gel gently for 30 min.

: *Make sure staining should be in dark.*

17. Rinse the gel and the staining tray 3 times with distilled water.

: *3 times washing is very important to remove all silver nitrate solution.*

18. To the 100 ml of developer add 125 μl of formaldehyde solution. Transfer the developer/formaldehyde solution to the staining tray and shake the tray gently in an indirect light.

: *Cover the container in aluminium foil.*

When the solution turns yellow to dark black precipitate become noticeable; replace the developer/formaldehyde solution with a second batch of 100 ml of the same solution. Continue to shake the gel in indirect light. Monitor the appearance of bands and background. When the ratio of the signal to the noise is high, remove the second batch of developer/formaldehyde solution.

: *Timing of adding fresh developer is very important, delay can cause the black background of the gel.*

19. Add 250 ml of 3% acetic acid to the staining tray immediately. Shake the gel gently for 5 min. Remove the 3% acetic acid and wash the gel with 10% ethanol. Remove the ethanol and store the gel for 2 min in a fresh batch of 10% ethanol. You can clearly see the bright polymorphic bands in a white background.

: *Timing of adding acetic acid is also very important, delay can cause the black background of the gel.*

: *Gel can be stored at this stage for several weeks in 10% ethanol.*

20. Scan the gel in gel scanner.

## 4 Timing

1. Genomic DNA isolation (2–3 h).
2. Polymerase chain reaction using microsatellite markers (3–4 h).
3. Non-denaturing polyacrylamide gel electrophoresis for the separation of the bands, steps 1–20 (5–6 h).

## 5 Troubleshooting

Troubleshooting advice can be found in Table 2.

### 51 Advantage of silver staining of DNA over other staining methods

1. Silver staining avoids radioactive handling, delays from development times and waste disposal issues and offers similar sensitivity to autoradiography.
2. Under normal light image development and visualization are done. Therefore, the procedure can be performed entirely at the lab bench without the need for UV illumination facilities and darkroom.
3. Because silver is deposited directly on the molecules within the transparent gel matrix, the image is resolved with the best possible sensitivity and detail.
4. For creating a permanent record of the original material, silver stained gels can be dried onto a semi-rigid plastic backing film. They can also be stored for a long time without breaking, removing the need and added expense of printing and photography. In addition, the preserved gel contained real stained DNA bands that can be extracted, amplified, cloned and DNA-sequenced.

#### 5.2 Anticipated results

**Fig. 1:**
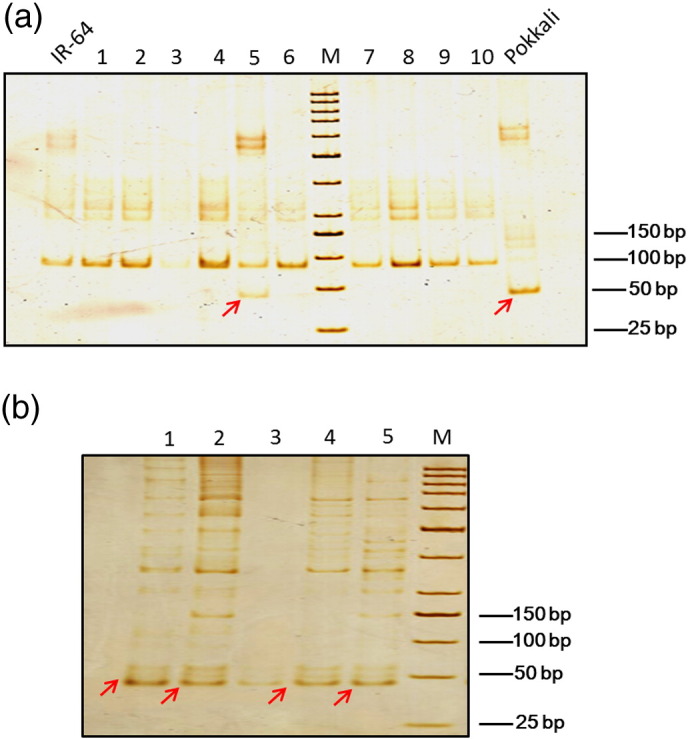
PAGE of SSR markers of salt tolerant mutant lines of ***Oryza sativa*** and ***Brassica juncea***. (a). PAGE of mutant lines(1–10) of rice amplified with specific SSR marker (details not given) at Tm 64 °C. Pokkali is a wild type salt tolerant cultivar. (b). PAGE of mutant lines (1–5) of *Brassica juncea* amplified with specific SSR marker (details not given) at Tm 66 °C. Lines 1, 2, 4 and 5 are salt tolerant lines. Red arrow indicates polymorphism.

**Fig. 2:**
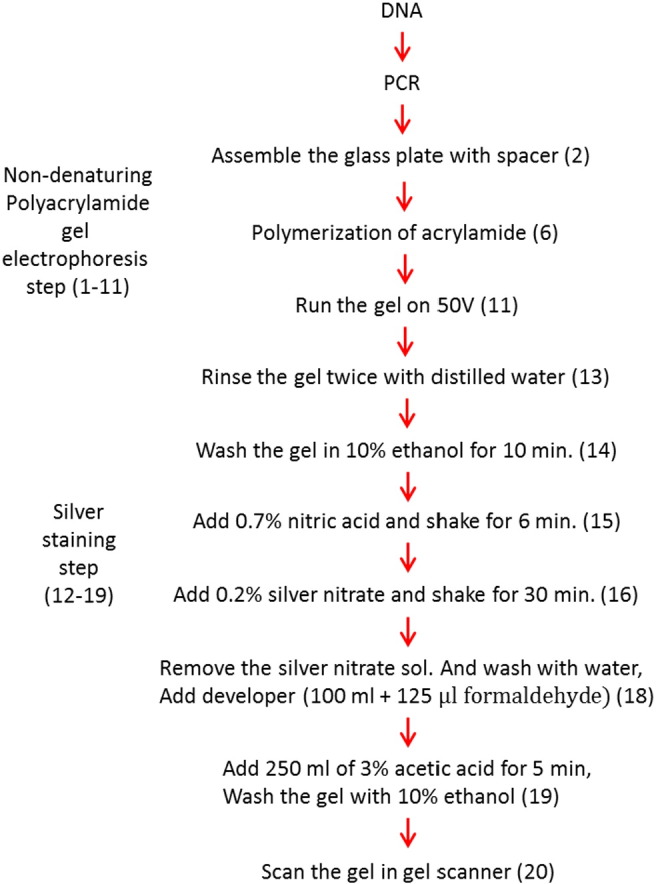
Flow diagram to illustrate the major steps of the way to assess the small polymorphic bands. Each step, which is shown in parentheses, corresponds to the step in the Procedure section.

To check the polymorphic bands for salinity tolerance by silver staining method, PAGE gels were run as described in the procedure (steps 1–20). In case of *O.* *sativa*, gel was loaded with thirteen sets of lanes consisting of a DNA size marker and twelve lanes of amplification products from a SSR primer already tested as polymorphic for the salinity tolerant Pokkali parent line (Fig. 1a). In case of *B.* *juncea*, gel was loaded with six sets of lanes consisting of a DNA size marker and five lanes of amplification products from a SSR primer already tested as polymorphic for the salinity tolerant *B.* *juncea* parent line (Fig. 1b). Gel development was stopped when the image reached optimal image contrast (as judged by the eye).

#### 5.3 Application

A valuable application of this procedure has on the detection of small nucleic acids (20–50 nucleotide length) that are polymorphic in nature for various phenotypic variations (in this case it was salinity stress tolerance). When compared with the 11 alternative treatment, as listed in Table 1, the results obtained by our method were less time consuming and easy to implement on normal basic facilities available.

Here we present an updated and optimized simple and efficient way to detect small polymorphic bands in plants.

## Competing interests

The authors declare that they have no competing interest.

## Figures and Tables

**Table 1:** Comparison of silver staining protocols from this method with others.

Step	Method 1 Morrissey [4]	Method 2 Bassam et al. [5]	Method 3 Sanguinetti et al. [6]	Method 4 Creste et al. [7]	Method 5Qu et al. [8]	Method 6 Modification of Benbouza et al. [9]	Method 7 Ji et al. [10]	Method 8 Zhang et al. [11]	Method 9 Han et al. [12]	Method 10 Byun et al. [13]	Method 11 An et al. [14]	Method 12 Our procedure
Prefix	50% methanol, 10% acetic acid (10 min) 5% methanol, 7% acetic acid (30 min)	–	–	1.5% nitric acid (3 min)	–	–	–	–	–	–	–	–
Fixation	10% glutaraldehyde (30 min)	10% acetic acid(20 min)	10% acetic acid 0.5% acetic acid(3 min)	10% ethanol, 1% acetic acid (10 min)	–	10% absolute ethanol 0.5% acetic acid (5 min)	–	–	–	–	–	–
Rinse	H_2_O (overnight) H_2_O (30 min) H_2_O (2 h)	H_2_O (2 min) 3 times	–	H_2_O (1 min)	–	–	–	H_2_O (10 s) Two times	–	–	–	H_2_O (5 s) 2 times
Soaking	5g/ml dithiothreitol (30 min)	–	–	–	–	–	–	–	–	–	–	–
Impregnation	0.1% AgNO_3_ (30 min)	0.1% AgNO_3_ 1.5 ml 37% HCOH (30 min)	2% AgNO_3_ (5 min)	2% AgNO_3_ (20 min)	25% ethanol, 1% nitric acid, 2% AgNO_3_ (5–10)	1.5% AgNO_3_, 1.5 ml 37% HCOH (22–24 °C) (6–7 min)	0.1% AgNO_3_ (10–15 min)	0.1% AgNO_3_ (4 °C) (15 min)	1% nitric acid 0.1% AgNO_3_ (5 min)	10% ethanol, 0.5%acetic acid, 0.2% AgNO_3_ (3–20 min)	5% ethanol, 1% nitric acid, 0.1% AgNO_3_ (5 min)	10% ethanol 0.7% nitric acid 0.22% AgNO_3_, (Room temp.) (5–30 min)
Rinse	H_2_O One time	H_2_O, 20 s, optional	Distilled H_2_O, 1 s	H_2_O (30 s) Two times	H_2_O (3 min)	Distilled H_2_O, (10 s) 3 times	H_2_O (5 s) Two times	2% NaOH, 0.01% HCOH, (10 s)	H_2_O (5 s) Two times	H_2_O (5 s) One times	H_2_O (10 s)	–
Development	50 l 37% formaldehyde + 100 ml 3% Na_2_CO_3_ 3–5 min	3% Na_2_CO_3_, 1.5 ml 37% HCOH; 2 mg Na_2_SO_3_.5H_2_O (10 °C) (2–5 min)	1.5% NaOH, 2 ml 37% HCOH (5 min)	3% Na_2_CO_3_, 1.5 ml 0.54 ml 37% HCOH (4-7 min)	3% Na_2_CO_3_, 0.2% HCOH, (2–5 min)	1.5% NaOH, 2 ml 37% HCOH (22–24 °C) (3–5 min)	0.04% Na_2_CO_3_, 0.2% HCOH, (5–6 min)	2% NaOH, 0.01% HCOH, (2-3 min)	2% NaOH, 0.04% NaCO_3_, 0.0025% EBT, 0.15% HCOH (5 min)	3% NaOH, 0.1% HCOH (55 °C) (5–10 min)	1.3% NaOH, 0.65% NaCO_3_, 0.4% HCOH; (2–3 min)	Per liter: 22.9 g Na_2_CO_3_, 1.25 ml 37% HCOH, 2 mg Na_2_SO_3_.5 H_2_O (4 °C) (3–5 min)
Stop	5 ml of 2.3 M citric acid	10% acetic acid(10 °C) (5 min)	–	5% acetic acid (5 min)	10% acetic acid,(2–5 min)	0.5% acetic acid 10% absolute ethanol (2 min)	H_2_O (5 s) Two times	H_2_O (5 s) Two times	2.5% ampicillin (5 s)Two times	10% ethanol, 0.5%acetic acid (1 min)	5% ethanol, 1% nitric acid (1 min)	250 ml of 3% acetic acid (4 °C) 10% ethanol 5 min

**Table 2:** Troubleshooting.

Problems	Cause	Solutions
No amplification in PCR	Annealing temperature problem	Varies the Tm from 56°–66°
No polymorphic bands	Primers are not polymorphic	Use different sets of markers
No bands at all	Using acetic acid as a fixative	Avoid using acetic acid as a fixative given in other methods as it stops the silver nitrate activity.
Bent band	High voltage and less buffer	Regularly add buffer to the top of gel and run gel as low voltage as possible
Broken bands	Gel pieces in the well	Clean the well properly before running the gel
Black background	Touching of the gel by naked hand and not washing gel properly as remaining pieces of gel can cause that.	Use cloves during silver staining, clean the gel properly.

## References

[1] Mitchell S.E., Kresovich S., Jester C.A., Hernandez C.J., Szwec-McFadden A.K. (1997). Application of multiplex PCR and fluorescence-based, semi-automated allele sizing technology for genotyping plant genetic resources. Crop Sci..

[2] Lagoda P.J.L., Dambier D., Grapin A., Baurens F.C., Lanaud C., Noyer J.L. (1998). Nonradioactive sequence-tagged microsatellite site analyses: a method transferable to the tropics. Electrophoresis.

[3] Merril C.R., Goldman D., Sedman S.A., Ebert M.H. (1981). Ultrasensitive stain for proteins in polyacrylamide gels shows regional variation in cerebrospinal fluid proteins. Science.

[4] Morrissey J.H. (1981). Silver stain for proteins in polyacrylamide gels: a modified procedure with enhanced uniform sensitivity. Anal. Biochem..

[5] Bassam B.J., Caetano-Anollés G., Gresshoff P.M. (1991). Fast and sensitive silver staining of DNA in polyacrylamide gels. Anal. Biochem..

[6] Sanguinetti C.J., Dias Neto E., Simpson A.J. (1994). Rapid silver staining and recovery of PCR products separated on polyacrylamide gels. Biotechniques.

[7] Creste S., Tulmann Neto A., Figueira A. (2001). Detection of single sequence repeat polymorphisms in denaturing polyacrylamide sequencing gels by silver staining. Plant Mol. Biol. Rep..

[8] Qu L., Li X., Wu G., Yang N. (2005). Efficient and sensitive method of DNA silver staining in polyacrylamide gels. Electrophoresis.

[9] Benbouza H., Jacquemin J.M., Baudoin J.P., Mergeai G. (2006). Optimization of a reliable, fast, cheap and sensitive silver staining method to detect SSR markers in polyacrylamide gels. Biotechnol. Agron. Soc. Environ..

[10] Ji Y.T., Qu C.Q., Cao B.Y. (2007). An optimal method of DNA silver staining in polyacrylamide gels. Electrophoresis.

[11] Zhang C.L., Wang Y., Chen H., Lan X.Y., Lei C.Z. (2007). Enhance the efficiency of single-strand conformation polymorphism analysis by short polyacrylamide gel and modified silver staining. Anal. Biochem..

[12] Han Y.C., Teng C.Z., Hu Z.L., Song Y.C. (2008). An optimal method of DNA silver staining in polyacrylamide gels. Electrophoresis.

[13] Byun S.O., Fang Q., Zhou H., Hickford J.G.H. (2009). An effective method for silver staining DNA in large numbers of polyacrylamide gels. Anal. Biochem..

[14] An Z.W., Xie L.L., Cheng H., Zhou Y., Zhang Q., He X.G., Huang H.S. (2009). A silver staining procedure for nucleic acids in polyacrylamide gels without fixation and pretreatment. Anal. Biochem..

[15] Fulton T.M., Chunwongse J., Tanksley S.D. (1995). Microprep protocol for extraction of DNA from tomato and other herbaceous plants. Plant Mol. Biol. Report..

[16] Kang T.J., Loc N.H., Jang M.O., Jang Y.S., Yang M.S. (2003). Expression of the B subunit of *E. coli* heat-labile enterotoxin in the chloroplasts of plants and its characterization. Transgenic Res..

